# ACE2 and SARS-CoV-2 Infection Risk: Insights From Patients With Two Rare Genetic Tubulopathies, Gitelman's and Bartter's Syndromes

**DOI:** 10.3389/fmed.2021.647319

**Published:** 2021-05-04

**Authors:** Lorenzo A. Calò, Matteo Rigato, Luca Sgarabotto, Lisa Gianesello, Giovanni Bertoldi, Verdiana Ravarotto, Paul A. Davis

**Affiliations:** ^1^Dialysis and Transplantation Unit, Department of Medicine, Nephrology, University of Padova, Padua, Italy; ^2^Department of Nutrition, University of California, Davis, Davis, CA, United States

**Keywords:** SARS-CoV-2, ACE2, Gitelman syndrome, Bartter syndrome, angiotensin conversing enzyme inhibitor, angiotensin II receptor blockers

## Abstract

COVID-19 is spreading globally with the angiotensin converting enzyme (ACE)-2 serving as the entry point of SARS-CoV-2 virus. This raised concerns how ACE2 and the Renin-Angiotensin (Ang)-System (RAS) are to be dealt with given their roles in hypertension and their involvement in COVID-19's morbidity and mortality. Specifically, increased ACE2 expression in response to treatment with ACE inhibitors (ACEi) and Ang II receptor blockers (ARBs) might theoretically increase COVID-19 risk by increasing SARS-CoV-2 binding sites. However, ACE2 is part of the protective counter-regulatory ACE2-Ang1-7-MasR axis, which opposes the classical ACE-AngII-AT1R regulatory axis. We used Gitelman's and Bartter's syndromes (GS/BS) patients, rare genetic tubulopathies that have endogenously increased levels of ACE2, to explore these issues. Specifically, 128 genetically confirmed GS/BS patients, living in Lombardia, Emilia Romagna and Veneto, the Northern Italy hot spots for COVID-19, were surveyed *via* telephone survey regarding COVID-19. The survey found no COVID-19 infection and absence of COVID-19 symptoms in any patient. Comparison analysis with the prevalence of COVID-19 in those regions showed statistical significance (*p* < 0.01). The results of the study strongly suggest that increased ACE2 does not increase risk of COVID-19 and that ACEi and ARBs by blocking excessive AT1R-mediated Ang II activation might favor the increase of ACE2-derived Ang 1-7. GS/BS patients' increased ACE2 and Ang 1-7 levels and their characteristic chronic metabolic alkalosis suggest a mechanism similar to that of chloroquine/hydroxychloroquine effect on ACE2 glycosylation alteration with resulting SARS-COV-2 binding inhibition and blockage/inhibition of viral entry. Studies from our laboratory are ongoing to explore GS/BS ACE2 glycosylation and other potential beneficial effects of BS/GS. Importantly, the absence of frank COVID-19 or of COVID-19 symptoms in the BS/GS patients cohort, given no direct ascertainment of COVID-19 status, suggest that elevated ACE2 levels as found in GS/BS patients at a minimum render COVID-19 infection asymptomatic and thus that COVID-19 symptoms are driven by ACE2 levels.

## Introduction

The ongoing SARS-CoV-2 disease (COVID-19) global pandemic has resulted in substantial morbidity and mortality with Italy being unfortunately one of Europe's COVID-19 hot spots. The identification of angiotensin converting enzyme 2 (ACE2) as the entry point of the SARS-CoV-2 virus has led to an intense scrutiny of Renin-Angiotensin System (RAS) and its roles as part of SARS-CoV-2's infection process as well as RAS's role in the pathophysiology associated with COVID-19 infection. The involvement of ACE2 has given rise to conflicting suggestions as to how ACE2 specifically, and RAS in general, should inform the treatment of COVID-19 (1, 2). The conflicting suggestions regarding the use or discontinuation of ACE inhibitors (ACEi) and angiotensin (Ang) II receptor blockers (ARBs) arose as these drugs are held to increase ACE2 expression, thereby raising the chance of COVID-19 infection *via* the increase in SARS-CoV-2 binding sites (1). The risk of COVID-19 posed by this mechanism must, however, be balanced against the widespread use and utility of these drugs to control hypertension as well as the increasingly recognized counter-regulatory, protective role of the ACE2-Ang 1-7-MasR axis in counterbalancing classical ACE-Ang II-type 1 receptor (AT1R) regulatory axis of RAS (3).

The multiplicity of ACEi and ARBs in use and their multiple off-target effects have made it difficult to draw conclusions with respect to their as well as ACE2's role in COVID-19. We have previously reported that our cohort of Gitelman's and Bartter's syndromes (GS/BS) patients have endogenously increased levels of ACE2 and Ang 1-7 (4). Briefly, GS/BS are rare tubulopathies caused by defects in specific kidney transporters and ion channels genes, which result in high Ang II and aldosterone levels and activation of RAS yet have normotension or hypotension. Moreover, GS/BS patients exhibit hyporesponsiveness to pressors as well as activation of anti-atherosclerotic and anti-remodeling defenses, reduction of oxidative stress and inflammation. Of particular interest is that GS/BS patients have increased and correlated levels of ACE2 and Ang 1-7, compared to hypertensive patients and healthy subjects, which may be useful in assessing the potential role of RAS and the ACE2/Ang 1-7 axis in COVID-19 (4, 5).

We report here results of a survey regarding COVID-19 amongst our 128 GS/BS patient cohort who live in Lombardia, Emilia Romagna, and Veneto, the hot spot regions of COVID-19 pandemic in Northern Italy.

## Methods

### Population and Survey Design

One hundred twenty-eight subjects were enrolled in this study. Inclusion criteria were a genetically confirmed diagnosis of GS or BS and residence in one of the COVID-19 hot-spot regions in Northern Italy: Lombardia, Emilia Romagna, and Veneto.

The study data were collected *via* a telephone survey conducted on April 24, 2020. The survey collected demographic characteristics and specifically asked COVID-19-related questions. These included history of recent travel in endemic areas, direct contacts with COVID-19 confirmed positive cases, and symptoms of SARS-CoV-2 infection (fever, cough, sore throat, asthenia, dyspnea, myalgia, anosmia/hyposmia, and ageusia). Subjects were asked to restrict their answers to the COVID-19-related questions to the period from January 2020 to the date of the survey, April 2020 (the first Italian COVID-19 case was identified on January 2020). All the subjects were following their usual GS or BS treatment, which included potassium and magnesium supplements.

### Patient Consent

Due to the infection risks related to the pandemic and the resulting government lockdown measures adopted, it was impossible to obtain written signed informed consent. Therefore, verbal consent to study participation was obtained at the start of the phone call. Participants were informed that the survey would use anonymized data and that a written study consent form will be provided upon their first visit to the hospital. In case of minors, patients' consents and information was provided by the parents. This study design was approved by the Ethics Committee.

### Statistical Analysis

The reported COVID-19 prevalence in Northern Italy was obtained from data by the Italian government Civil Protection organization on SARS-CoV-2-positive individuals on June 26, 2020 (6); estimated true COVID-19 prevalence in Northern Italy was assessed from prevalence data published by Signorelli et al. (7). Exact 95% confidence intervals (95% CI) for prevalence were calculated using the Clopper-Pearson method. Fisher's exact test for count data was used to compare data. All the analyses were performed with the R program, version 4.0.2 (8).

## Results

Demographic and clinical characteristics of the sample and COVID-19-related data are summarized in Table 1.

**Table 1:** Demographics and clinical characteristics of patients.

**Variable**	**Number**
Gitelman's syndrome	116
Bartter's syndrome	12
Age—years (±SD/SE)	37.04 ± 17.8
Age range	7–75
Gitelman's syndrome	
Under 17	0
Under 40	60
Bartter's syndrome	
Under 17	7
Under 40	5
Gender	
Male	28
Female	100
Region of residence	
Lombardia	32
Emilia romagna	6
Veneto	90
Symptoms—number of subjects (%)	
Fever	0 (0%)
Cough	0 (0%)
Sore throat	0 (0%)
Asthenia	0 (0%)
Dyspnea	0 (0%)
Myalgia	0 (0%)
Anosmia/hyposmia	0 (0%)
Ageusia	0 (0%)
Travels in endemic areas	0 (0%)
Contacts with COVID-19 confirmed positive cases	0 (0%)

Official COVID-19 prevalence estimation on June 26, 2020 reported 239,961 positive cases of COVID-19 out of the general population of 60.36 million people, which made the population COVID-19 prevalence of 0.40% 95% CI (0.40–0.40%) (6). A breakdown by region of positive cases showed 93,587 in Lombardia (population 10.06 million), 28,393 in Emilia Romagna (population 4.459 million), and 19,262 in Veneto (population 4.906 million). The resulting total prevalence combining these regions was 0.73% (95 CI 0.72–0.73%). Initially, the prevalence of COVID-19-positive Italians was held to be around 0.40% based on SARS-CoV-2 cases in the hot spots in Northern Italy (6). However, several retrospective analyses have recently found that this relatively low level is not accurate due to reporting bias, such as, the low number of swabs performed, especially in the early phases of the pandemic (7, 9–11). A more realistic assessment estimated the nationwide Italian COVID-19 prevalence to be 10-fold higher, while prevalence in the Northern Italy “hot spots” was even higher (7). The analysis performed by Signorelli et al. (7) found that positive cases could be estimated in 1,337,980 in Lombardia, 307,671 in Emilia Romagna, and 98,120 in Veneto. Using these data, the total “hot spot regions” of COVID-19 prevalence is 8.96% (95% CI 8.96–8.99%).

The COVID-19-related survey data (Table 1) found a complete absence of COVID-19 symptoms, making the number of positives in the GS/BS patients cohort 0.00% (95% CI 0.00–3.62%). Using the survey population size (*n* = 128) and assuming that the surveyed population represented a random sample of the “hot spot regions,” the probability of obtaining zero COVID-19-infected subjects upon surveying 128 subjects using the official prevalence of the “hot spot regions” was not significant (*p* = 0.789). However as noted, using a more realistic prevalence based on the analysis of Signorelli et al. (7), the probability becomes statistically significant (*p* < 0.01).

None of the participants reported any close contact with subjects affected by COVID-19. GS/BS patients were mainly females (100/128). The Italian ISS COVID-Translational research group reported a similar percentage of COVID-19-positive subjects between the two genders at the time of this study (12). Using Signorelli's model (7), to calculate a more accurate COVID-19 prevalence and again assuming the number of population positives is equally distributed between genders, reports have shown no real differences between male and female COVID-19 infection rates (13, 14) and results in an estimated 871,886 female positives. And 51.2% of the population in Lombardia, Emilia Romagna, and Veneto were female, resulting in a female COVID-19 prevalence of 8.75% (CI 95% 8.74–8.77). Using these to statistically analyze the results for the surveyed GS/BS females (0/100), it was found that the absence of COVID-19-related symptoms was statistically significant (*p* = 0.0026). However, doing the same analysis on GS/BS males found an absence of COVID-19-related symptoms was not statistically significant, *p* = 0.167. This is most likely due to the small number (*n* = 28) of male GS/BS who participated in the survey.

## Discussion

The COVID-19 pandemic has focused interest on the RAS, as not only is it an integral part of SARS-CoV-2's infection process but also plays a major role in lung injury, a major cause of COVID-19's morbidity and mortality. The current state of our understanding of RAS and SAR-CoV-2 has led to conflicting suggestions regarding how RAS and its role in COVID-19 should inform the handling of COVID-19-positive patients with hypertension. An area of major disagreement arises from the fact that ACEi or ARBs potentially upregulate ACE2, the entry point for SARS-CoV-2, which by increasing ACE2 might increase SARS-CoV-2 infection rate (1). This approach has been criticized (15), particularly given the extensive use and benefits of ACEi or ARBs to treat hypertension. In fact, there are suggestions that ARBs might be beneficial in COVID-19 (2), as SARS-CoV-2 causes ACE2 downregulation slowing the Ang II conversion to the vasodilatory (2), anti-inflammatory and anti-atherosclerotic Ang 1-7 (16–18), while the use of ARBs would be beneficial by blocking the excessive Ang II AT1R-mediated Ang II activation, upregulating ACE2 activity, and increasing Ang 1-7 levels.

The potential protective role of ACE2 in SARS-CoV-2 infection-induced acute respiratory distress syndrome (ARDS), the major cause of COVID-19 mortality as well as other risk factors, such as hypertension, diabetes, and cardiovascular disease that are linked to COVID-19 morbidity and mortality, have been recently reviewed (19). In addition, Abedi et al. (20) have recently reviewed the relationship between Rho kinase (ROCK), acute lung injury and Acute Respiratory Distress Syndrome, and the beneficial effect of ROCK inhibitors on lung injury, also noting the increased activity and levels of ACE2 caused by ROCK inhibitors (21). Our studies in GS/BS to explore and better define the human RAS and RhoA/ROCK systems (5, 22, 23) provide further background as to the protective effects of increased levels of ACE2 along with ROCK inhibition and how those might be of use against SARS-CoV-2 infection-induced respiratory complications. Specifically, GS/BS patients have an activated RAS and high Ang II levels, yet blunted Ang II-mediated cardiovascular effects and normotension or hypotension. Moreover, they have increased and correlated levels of both ACE2 and Ang 1-7 accompanied by activation of anti-inflammatory, antiapoptotic, antiproliferative, and anti-atherosclerotic defenses; reduced oxidative stress (4, 5); and blunted Rho kinase signaling (5, 24). These patients have also upregulated regulator of G-protein signaling (RGS)-2, which acts as a negative regulator for Ang II signaling *via* AT1R, which includes the Ang II-mediated activation of the RhoA/ROCK system (5).

There are other characteristics of GS/BS patients that potentially beneficially affect ACE2. GS/BS patients have a characteristic chronic metabolic alkalosis, which may have direct effects on ACE2 itself similar to that of chloroquine/hydroxychloroquine (CQ). Vincent et al. (25) have reported that CQ interfered with terminal glycosylation of ACE2, the SARS-CoV (SARS-associated coronavirus) receptor, which shares with SARS-CoV-2 the same ACE2 receptor binding as the means to infect cells (26). CQ's negative effects on SARS-CoV's receptor binding, while inhibiting infection (25), left unaltered ACE2 membrane expression but impaired ACE2's terminal glycosylation *via* effects on Trans Golgi Network (TNG)/post-Golgi pH homeostasis (25). The characteristic metabolic alkalosis present in GS/BS patients could reproduce the same, pH dependent, effect(s) on ACE2 glycosylation, thereby impacting not only the GS/BS phenotypes (27), but also perhaps blocking/inhibiting SARS-CoV-2 binding and thereby reducing COVID-19 infections (25).

The clear centrality of ACE2 and therefore of RAS to COVID-19 provide the rationale for our prevalence of COVID-19 survey in GS/BS patients as they all share a characteristic endogenously increased ACE2, ROCK inhibition, and activation of anti-inflammatory and antioxidant defenses (4, 5). Our survey found no COVID-19 infection in the 128 interviewed patients of our cohort, all of whom reside in one of the Italian hot spots of COVID-19 pandemic. This absence of COVID-19 from our patient cohort was shown to be significant when statistically compared to the adjusted prevalence of COVID-19 in the general northern Italian population (7), suggesting that increased ACE2 and reduced ROCK activity likely provide benefits in terms of reduced risk of COVID-19, such as those we found in GS/BS. In fact, Cheng et al. argue that we should focus on increasing ACE2 as a method of treating COVID-19 (19), and studies with the use of human recombinant soluble ACE2 (hrsACE2) (28) go toward this direction. hrsACE2 has already passed through phase I and II clinical trials (NCT00886353, NCT01597635) for acute respiratory distress syndrome and has received regulatory approval (NCT04335136) for continued study in the fight against COVID-19 (29). Others have suggested that RAS inhibitors might be beneficial for COVID-19 induced acute kidney injury (30). Our results may represent an underestimate of the prevalence of COVID-19 in our cohort given that there was no effort to actually test every patient for COVID-19. However, accepting that there were missed COVID-19 infected patient(s), this would then suggest that the ACE2 relayed effects of GS/BS renders COVID-19 infections asymptomatic, and therefore COVID-19 symptoms are likely driven by ACE2 levels. In summary, our survey on 128 GS/BS patients found no diagnosed COVID-19 or COVID-19-related symptoms. Although limited to a small cohort size given both the rare nature of the syndromes and to a relative short period of time, the survey's results showing a zero prevalence of COVID-19 proved to be statistically significant when using the adjusted estimated prevalence of COVID-19 observed in the general population of the hot spot regions of Northern Italy for COVID-19 in the same time period (7). The increased level of ACE2 and the inhibited ROCK activity of GS/BS (4, 5, 24), both credited with protective effects against COVID-19 infection, may provide a mechanistic basis for our findings. GS/BS patients' characteristic chronic metabolic alkalosis may have altered ACE2's terminal glycosylation in the TGN/endosome system (27) by blocking its acidification necessary for the ACE2 glycosylation process, thereby blocking/inhibiting SARS-CoV-2 binding and resulting COVID-19 disease. Studies from our laboratory are ongoing to investigate ACE2 glycosylation state as well as other potential GS/BS-related benefits with respect to COVID-19.

## Data Availability Statement

The raw data supporting the conclusions of this article will be made available by the authors, without undue reservation.

## Ethics Statement

Ethical review and approval was not required for the study on human participants in accordance with the local legislation and institutional requirements. Written informed consent for participation was not provided by the participants' legal guardians/next of kin because due to the infection risks related to the pandemic and the resulting government lockdown measures adopted, it was impossible to obtain written signed informed consent. Therefore, verbal consent was obtained at the start of the phone call consenting to study participation and use of anonymized data (immediate consent), according to information filed with the Ethics Committee. Participants were informed that a written study consent form would be provided upon their first visit to the hospital (deferred consent). In case of minors, patients' consent was obtained from parents and for those patients under 10 information were provided by the parents.

## Author Contributions

LC and PD conceived and designed the study and wrote the final manuscript. LG acquired and analyzed the data. MR, LS, VR, and GB interviewed the patients and contributed to drafting the manuscript. All authors read and approved the manuscript.

## Conflict of Interest

The authors declare that the research was conducted in the absence of any commercial or financial relationships that could be construed as a potential conflict of interest.
